# The Effects of a Cardiac Rehabilitation Program on Endothelial Progenitor Cells and Inflammatory Profile in Patients with Chronic Heart Failure of Different Severity

**DOI:** 10.3390/jcm12206592

**Published:** 2023-10-18

**Authors:** Christos Kourek, Alexandros Briasoulis, Eleftherios Karatzanos, Virginia Zouganeli, Katherina Psarra, Maria Pratikaki, Androula Alevra-Prokopiou, John Skoularigis, Andrew Xanthopoulos, Serafim Nanas, Stavros Dimopoulos

**Affiliations:** 1 Clinical Ergospirometry, Exercise & Rehabilitation Laboratory, 1st Critical Care Medicine Department, Evangelismos Hospital, National and Kapodistrian University of Athens, 10676 Athens, Greece; chris.kourek.92@gmail.com (C.K.); lkaratzanos@gmail.com (E.K.); sernanas@gmail.com (S.N.); 2 Department of Cardiology, 417 Army Share Fund Hospital of Athens (NIMTS), 11521 Athens, Greece; 3 Department of Clinical Therapeutics, Faculty of Medicine, Alexandra Hospital, National and Kapodistrian University of Athens, 11528 Athens, Greece; alexbriasoulis@gmail.com; 4 Division of Cardiovascular Medicine, Section of Heart Failure and Transplantation, University of Iowa Hospitals and Clinics, Iowa, IA 52242, USA; 5 Second Cardiology Department, Attikon University Hospital, Medical School, National and Kapodistrian University of Athens, 12462 Athens, Greece; virginia_noa@yahoo.gr; 6 Immunology and Histocompatibility Department, Evangelismos Hospital, 10676 Athens, Greece; kpsarra@outlook.com; 7 Clinical Biochemistry Department, Evangelismos Hospital, 10676 Athens, Greece; mpratikaki@yahoo.com (M.P.); aaprokopiou@hotmail.com (A.A.-P.); 8 Department of Cardiology, University Hospital of Larissa, 41334 Larissa, Greece; iskoular@gmail.com (J.S.); andrewvxanth@gmail.com (A.X.); 9 Cardiac Surgery Intensive Care Unit, Onassis Cardiac Surgery Center, 17674 Athens, Greece

**Keywords:** chronic heart failure (CHF), exercise training, endothelial progenitor cells (EPCs), functional capacity, inflammation, severity

## Abstract

Endothelial dysfunction and inflammation are common pathophysiological characteristics of chronic heart failure (CHF). Endothelial progenitor cells (EPCs) are recognized as useful markers of vascular damage and endothelial repair. The aim of this study was to investigate the effects of a cardiac rehabilitation program on EPCs and inflammatory profile in CHF patients of different severity. Forty-four patients with stable CHF underwent a 36-session cardiac rehabilitation program. They were separated into two different subgroups each time, according to the median peak VO_2_, predicted peak VO_2_, VE/VCO_2_ slope, and ejection fraction. EPCs, C-reactive protein (CRP), interleukin 6 (IL-6), interleukin 10 (IL-10), and vascular endothelial growth factor (VEGF) were measured. Flow cytometry was used for the quantification of EPCs. Mobilization of EPCs increased and the inflammatory profile improved within each severity group (*p* < 0.05) after the cardiac rehabilitation program, but there were no statistically significant differences between groups (*p* > 0.05). A 36-session cardiac rehabilitation program has similar beneficial effects on the mobilization of EPCs and on the inflammatory profile in patients with CHF of different severity.

## 1. Introduction

Chronic heart failure (CHF) is a clinical syndrome characterized by increased morbidity and mortality, along with a significant financial and social burden [1]. Common pathophysiological characteristics of CHF include endothelial dysfunction and inflammation [2]. Patients with CHF frequently present higher levels of inflammatory markers such as C-reactive protein (CRP) and interleukin 6 (IL-6) [3]. Circulating endothelial cells (CECs) and endothelial progenitor cells (EPCs) are recognized as useful markers of vascular damage and endothelial repair in response to vascular injury in cardiovascular diseases [4]. CECs are mature cells that have been shed from the lining of the vascular wall into the bloodstream [5], while EPCs are bone-marrow-derived endothelial cells that promote endothelial repair, neovascularization, and endothelial function [4]. Reduced levels of EPCs and CECs in patients with heart failure with reduced ejection fraction (HFrEF), compared to age-matched subjects without established cardiovascular disease, suggest that these cellular populations may be potential biomarkers of the cellular response to vascular injury [6].

Either a single bout of exercise or a structured exercise training program has been shown to increase the mobilization of EPCs and CECs in patients with HFrEF or heart failure with mildly reduced ejection fraction (HFmrEF) [7,8,9]. It has also been shown that a single session of maximal exercise increases the mobilization of EPCs and CECs in a similar way in CHF patients, regardless of the syndrome’s severity [10]. However, data regarding the impact of exercise training on the mobilization of EPCs and CECs in CHF patients of different severity, according to functional capacity indices, still remain limited.

We hypothesized that exercise training would have similar beneficial effects in patients with CHF of different severity, regardless of their previous functional capacity. The primary aim of the present study was to investigate the effects of a 3-month structured exercise training program on EPCs and CECs in patients with HFrEF and/or HFmrEF of different severity according to functional capacity indices. The secondary aim was to investigate potential differences in inflammatory indices in these patients.

## 2. Materials and Methods

This is a post hoc analysis of a previously published randomized controlled trial from our institute [9] (Ap. number: 117/3–7–2017) that investigated the mobilization of EPCs and CECs after exercise training in patients with CHF.

### 2.1. Patients

Patients from heart failure outpatient clinics were referred for assessment to the “Clinical Ergospirometry, Exercise and Rehabilitation Laboratory” of “Evangelismos Hospital”, Athens. The patients were fully informed about the structure and the potential benefits and/or risks of the cardiac rehabilitation program and were asked to sign an informed consent form.

The inclusion criteria were (i) stable CHF for at least 3 months under medication and (ii) a reduced or mildly reduced ejection fraction (EF ≤ 49%). The exclusion criteria were (i) severe valvulopathy, (ii) uncontrolled arterial hypertension, (iii) severe chronic obstructive pulmonary disease, (iv) severe peripheral angiopathy, (v) neuromuscular diseases, and (vi) contraindications for maximum CPET [11].

**Figure 1:**
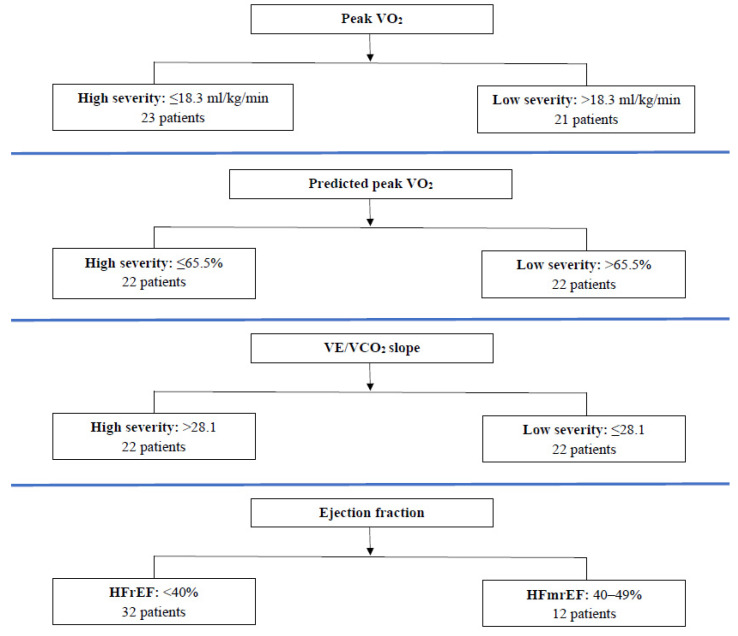
Severity groups of chronic heart failure patients according to the median values of peak VO_2_, predicted peak VO_2_, VE/VCO_2_ slope, and class of ejection fraction.

The patients were separated into 2 different subgroups each time, according to the median values of peak VO_2_, predicted peak VO_2_, VE/VCO_2_ slope, and class of EF (Figure 1).

### 2.2. Study Design

After the initial screening and assessment, 44 patients with CHF underwent a 36-session cardiac rehabilitation program, with 3 sessions per week. In the beginning, all of the patients performed a symptom-limited maximal cardiopulmonary exercise test (CPET) on an electromagnetically braked cycle ergometer in order to assess their functional capacity and CHF severity. Blood samples were collected before and after CPET. In the meantime, the patients underwent a 36-session exercise training program and, after the completion of the program, the CHF patients performed a final CPET so as to assess the impact of the exercise training program on their functional capacity. Blood samples were also collected before and after the final CPET.

The ramp symptom-limited maximal CPET on an electromagnetically braked cycle ergometer (Ergoline 800; SensorMedics Corporation, Anaheim, CA, USA) had a duration of 8–12 min and was performed according to Hansen et al.’s protocol [12]. The main breathing parameters that were calculated in patients through their gas exchanges were VO_2_, VCO_2_, and VE, as well as more specific variables such as resting VO_2_, VO_2_ at peak exercise (peak VO_2_), predicted VO_2_ at peak exercise (predicted peak VO_2_), and VE/VCO_2_ slope [13]. The endpoints of CPET were abnormal ECG rhythm at the monitor, dyspnea, or leg fatigue.

### 2.3. Exercise Training Protocol

The patients underwent a HIIT protocol as previously described in [9], which was a modified version of Wisløff et al.’s protocol [14], or HIIT combined with muscle training. The intensity was individually prescribed based on VO_2_–workload plots of the initial CPET [9]. Almost half of the patients performed strength training (2–3 sets, 10–12 repetitions, 60–75% of the 1-repetition maximum), including knee extension, knee flexion, and chest press exercises, while the other half performed balance and coordination exercises including narrow corridor walking, backward narrow corridor walking, and side walking on both sides [9]. Because of the fact that the initial cohort performed either aerobic exercise or aerobic exercise with muscle training, we performed an extra randomized stratification for the training protocol in the subgroups of our study, so that each subgroup would include an equal or almost equal number of patients performing only aerobic exercise or aerobic exercise combined with muscle training. In this way, we avoided a potential bias that would exist if there was no balance between the exercise training protocols.

### 2.4. Flow Cytometry Analyses for EPCs and CECs

Venous blood samples were collected in K3 ethylenediaminetetraacetic acid (K3-EDTA) tubes, while endothelial cellular populations were identified and quantified with the use of four-color flow cytometry within the first hour after the collection, based on Duda’s protocol [15] and our institution’s methodology as previously described in [9]. Each analysis with the flow cytometer included 10^6^ events. EPCs and CECs were expressed as medians (25th–75th percentiles) in absolute numbers of cells per 10^6^ enucleated cells.

### 2.5. Inflammatory Indices

C-reactive protein (CRP), interleukin 6 (IL-6), interleukin 10 (IL-10), and vascular endothelial growth factor (VEGF) were measured from the upper phase (plasma) of the total venous blood after the centrifugation [15,16]. Immunoturbidimetric assays were used for the in vitro quantitative determination of CRP in human plasma (Roche/Hitachi cobas c systems, Roche Diagnostics International Ltd.). Moreover, the BDTM CBA Human Soluble Protein Flex Set System was used to assess cytokine levels and VEGF [16]. Four-color flow cytometry was performed with a Navios (Beckman Coulter) flow cytometer. Values of IL-6, IL-10, and VEGF were expressed as medians (25th–75th percentiles) in pg/mL. Values of CRP were expressed as medians (25th–75th percentiles) in mg/dL.

### 2.6. Statistical Analyses

Patients were divided according to CHF severity based on CPET assessment, and the results are presented according to the severity groups. Normality of distribution was checked with the Shapiro–Wilk test. Variables are expressed as means ± standard deviations (SD) or medians (25th–75th percentiles). Paired two-sample Student’s *t*-tests were used to analyze differences in dependent parameters with normal distribution, while the Wilcoxon signed-rank test was used to analyze differences in nonparametric data within the total sample and within the severity groups. Independent-samples *t*-tests or the Mann–Whitney U test were used to analyze differences between independent parameters, based on the distribution of normality as appropriate. Chi-squared tests were employed to check for between-group differences in categorical variables at baseline. Unadjusted differences between severity groups were assessed with factorial analysis of variance (ANOVA) 2 × 2 × 2 (time × intervention × group). Linear regression analysis was performed between the absolute values and the percentages Δθ of each EPC and CEC subgroup after the cardiac rehabilitation program and the baseline values of functional capacity indices including peak VO_2_, predicted peak VO_2_, VE/VCO_2_ slope, EF, and age. Moreover, Spearman’s correlation coefficient was used in order to assess the direction and the magnitude of the association between the absolute and percentage differences of each endothelial cellular population and the values of CPET parameters and inflammatory indices. Due to the existence of a multiple comparison effect, statistical correction with Bonferroni’s test was performed in order to reduce the incidence of false positive findings. All tests were two-tailed, and the level of statistical significance was set at 0.05. Statistical analyses were performed with IBM SPSS 25 Statistics software (Armonk, NY, USA).

## 3. Results

The majority of the patients were mainly treated with diuretics, beta-blockers, aldosterone antagonists, or angiotensin-converting enzyme inhibitors. The patients’ compliance with the rehabilitation program was >80% in both groups. The patients did not differ in demographics between the two groups in each comparison, except for the variables for which they were separated (Table 1).

The mobilization of all EPC and CEC subgroups increased within each severity group (*p* < 0.05) after the cardiac rehabilitation program, but there were no statistically significant differences between groups (*p* > 0.05) in each comparison based on peak VO_2_ (Table 2), predicted peak VO_2_ (Table 3), VE/VCO_2_ slope (Table 4), and EF (Table 5).

Inflammatory status seemed to improve in CHF patients, as CRP decreased within each severity group and IL-10 increased in most comparisons (*p* < 0.05, Table 2, Table 3, Table 4 and Table 5). However, no differences between severity groups were observed in any of the comparisons (*p* > 0.05, Table 2, Table 3, Table 4 and Table 5). Similarly, neovascularization improved with the increase in VEGF in each severity group (*p* < 0.05, Table 2, Table 3, Table 4 and Table 5), albeit without significant differences between groups in each comparison (*p* > 0.05, Table 2, Table 3, Table 4 and Table 5).

As far as CPET indices are concerned, peak VO_2_, predicted peak VO_2_, VE/VCO_2_ slope, and peak work rate improved within each severity group in most comparisons after rehabilitation, but this improvement was similar between groups (*p* > 0.05, Table 2, Table 3, Table 4 and Table 5). Moreover, EF increased within each severity group in each comparison after the 36-session exercise training program (*p* < 0.05, Table 2, Table 3, Table 4 and Table 5), without differences between groups (*p* > 0.05, Table 2, Table 3, Table 4 and Table 5).

Finally, linear regression analysis did not show statistical significance between the absolute values and the percentages Δθ of each EPC and CEC subgroup after the cardiac rehabilitation program and the baseline values of functional capacity indices, including peak VO_2_, predicted peak VO_2_, VE/VCO_2_ slope, EF, and age (*p* > 0.05, Table 6). Correlations between the numeric and the percentage differences in the mobilization of endothelial cellular populations and the numeric differences in other cardiopulmonary exercise testing or blood sample variables after the cardiac rehabilitation program are demonstrated in Table S1 and Table S2, respectively.

## 4. Discussion

The new insight of our study in the literature is that a 36-session exercise training program has a similar beneficial effect in the enhancement of the mobilization of EPCs and CECs in patients with CHF, irrespective of their syndrome’s severity. Moreover, this beneficial effect seems to exist in other parameters of interest too, including inflammatory indices and neovascularization markers. This is the first time in the literature that a study has investigated the effects of HIIT, either alone or combined with muscle endurance training, in CHF patients of different severity according to functional capacity indices and different EF categories.

Common pathophysiological features of patients with CHF, as well as the exact mechanisms behind the repair of damaged vessels by EPCs, have already been described in a recent review from our institute [7]. VEGF promotes angiogenesis by inducing proliferation, differentiation, and chemotaxis of endothelial cells [17]. Indeed, in our study, the elevated plasma levels of the angiogenic growth factor VEGF in CHF patients were consistent with the increased number of circulating EPCs after exercise training. Our findings are in agreement with the findings of other studies where the numbers of EPCs and plasma levels of VEGF significantly increased after a cardiac rehabilitation program including aerobic exercise alone or combined with muscle endurance training in patients with CHF [18,19] or other cardiovascular diseases [20]. On the other hand, some studies have shown controversial results in CHF, as the increase in EPCs did not correlate with a statistically significant increase in VEGF [21]. This finding could be explained by the fact that our exercise training program included HIIT either combined with muscle endurance training or not, while other studies may include other exercise training protocols such as moderate-intensity continuous training (MICT). Indeed, HIIT seems to be superior to MICT in terms of functional capacity indices [22,23] and EPCs [24] in CHF patients. A recent meta-analysis by Fuertes-Kenneally L et al. [25] answered all of the above research questions by investigating (i) the effect of exercise-based cardiac rehabilitation on endothelial function, assessed by flow-mediated dilation in patients with HF, and (ii) whether HIIT is better than moderate-intensity training for improving endothelial function. The authors highlighted the beneficial effect of exercise training on FMD compared to the controls (MD+ = 3.09% (95% CI = 2.01, 4.17); *p* < 0.001) and the superiority of HIIT in terms of endothelial function compared to MICT (MD+ = 2.35% (95% CI = 0.49, 4.22); *p* = 0.013).

The most significant finding of our study is that the effect of exercise training on the mobilization of EPCs and CECs was beneficial for all CHF patients, irrespective of their severity. So far, there are no data in the literature regarding the effects of a cardiac rehabilitation program on EPCs as far as the comparison between CHF patients of different severity, according to functional capacity indices, is concerned. Only a single clinical study from our institute has examined the same research question, but after acute exercise, and it showed that the mobilization of EPCs and CECs increased after maximal exercise in CHF patients, but this increase was not associated with the syndrome’s severity [10]. This is the first study to answer this research question after a structured exercise training program.

As far as systemic inflammation is concerned, patients with CHF usually present elevated levels of inflammatory markers such as CRP [26] and IL-6 [27,28]. Regular exercise training has been shown to have anti-inflammatory effects in the skeletal muscles and vascular endothelium in CHF [29,30]. Previous studies from other institutes have demonstrated controversial findings regarding inflammatory indices. A significant decrease in CRP was demonstrated in patients with cardiovascular diseases other than CHF [20,31], but in CHF patients no difference in CRP was observed [21]. Similarly, previous studies showed that IL-6 remained unchanged in HF after exercise training [32,33]. A recent meta-analysis by Malandish A et al. [34] investigated the effects of concurrent, aerobic, and resistance exercise on markers of inflammation and vascular adhesion molecules including hsCRP, IL-6, IL-10, tumor necrosis factor alpha (TNF-α), and other markers such as soluble intercellular adhesion molecule-1 (sICAM-1), soluble vascular cell adhesion molecule-1 (sVCAM-1), fibrinogen, IL-1-b, IL-18, and E-selectin in patients with CHF. Exercise training was shown to improve inflammation and vascular adhesion markers by significantly reducing hsCRP (SMD −0.441 (95% CI: −0.642 to −0.240); *p* = 0.001), IL-6 (SMD −0.158 (95% CI: −0.303 to −0.013); *p* = 0.032), and sICAM-1 (SMD −0.282 (95% CI: −0.477 to −0.086), *p* = 0.005). In our study, an exercise training program reduced hsCRP and IL-6 levels and, in some instances, increased levels of IL-10, a major anti-inflammatory cytokine that reduces pathological hypertrophy and promotes cardiac remodeling [35,36]. The potential explanation is the superiority of HIIT compared to the other aerobic training protocols that were implemented in these studies. Indeed, HIIT seems to be superior to MICT in terms of functional capacity indices [22,23] in CHF patients. We suppose that HIIT could be superior in the improvement of the inflammatory profile too, but this has yet to be proven. Except for this improvement in the inflammatory profile of CHF patients, the new insight of our study is that this improvement was similar in all patients, both for those with higher and lower severity according to functional capacity indices.

Finally, the adherence of CHF patients to an exercise training protocol is another significant parameter of a cardiac rehabilitation program’s success. It is a matter of fact that a smaller number of sessions is usually followed by improved rates of adherence than a longer rehabilitation program for the same effect. A recent review by Collado-Mateo D et al. [37] investigated adherence to physical exercise in chronic patients and older adults in relation to the duration of the exercise intervention. It was shown that longer exercise interventions were related to lower adherence to the program, an outcome that may be associated with the need to maintain a homogeneous exercise routine throughout the entire exercise program [37]. This may cause some individuals to drop the program due to the lack of variety. However, in our study, the adherence rate was >80% in all groups despite the fact that the cardiac rehabilitation program consisted of 36 sessions. Most exercise training protocols investigating the effects of exercise on EPCs and CECs have a duration of at least 12 weeks [18,21,38,39,40]. Only a few studies have used a smaller number of sessions [19,33] with similar improvements in the number and function of EPCs; however, their adherence was not different from that of longer rehabilitation programs. Other characteristics of exercise, such as the type, the frequency, or the intensity, are not often reported as key factors to promote adherence. Specifically, as far as the frequency is concerned, studies have shown that exercise training once per week may lead to lower adherence, probably due to participants doubting its efficacy, the less frequent contact with the staff and peers, and the bias caused by the selection of physically active participants who may be unsatisfied with the low exercise frequency [41]. As a result, more exercise sessions within a week—for instance, three sessions—would seem to be the most appropriate frequency for a successful rehabilitation program.

Small sample size may be an important limitation of this study for some specific indices, other than EPCs, CECs, and inflammatory markers. Although power analysis was performed and the results are valid for cellular endothelial populations and cytokines, some specific between-group comparisons may have been too underpowered to reach definite conclusions and generate the results for other parameters of interest. Moreover, another possible limitation could be the different number of samples in each comparison for each variable. In the first three comparisons for CPET indices, we separated the total sample according to the median value of each variable for each analysis, so that the severity groups would be almost equal each time. The reason for this was that the functional capacity of our patients was moderate–high, and we had only a few patients with low functional capacity. As a result, we could not separate the sample into three groups of low, moderate, and high functional capacity. For the ejection fraction, the samples were not equal due to the fact that HF categories are well established and we compared HFmrEF and HFrEF. Finally, a multiple comparison effect that could arise from our analyses as a potential bias was corrected with the Bonferroni statistical test.

## 5. Conclusions

Our study suggests that a 36-session cardiac rehabilitation program has similarly beneficial effects on the mobilization of EPCs and CECs, as well as on inflammatory indices and indices of neovascularization, in patients with CHF of different disease severity, according to functional capacity indices and EF. The potential mechanisms still remain under investigation. More RCTs with greater numbers of CHF patients are required in order to confirm these significant results, reveal the pathophysiology, and further understand the clinical relevance of endothelial cellular populations in CHF.

## Figures and Tables

**Table 1:** Baseline demographic characteristics and cardiopulmonary exercise testing indices of patients with chronic heart failure of different severity enrolled in the cardiac rehabilitation program, according to peak VO_2_, predicted peak VO_2_, VE/VCO_2_, and EF.

Demographics	Peak VO_2_ (mL/kg/min)		Predicted Peak VO_2_ (%)		VE/VCO_2_		Ejection Fraction (%)	
	≤18.3	>18.3	≤65.5	>65.5	>28.1	≤28.1	<40	40–49
Number of patients (N)	23	21	22	22	22	22	32	12
Gender (*males*/*females)*	17/6	18/3	16/6	19/3	17/5	18/4	26/6	9/3
Age (years) ^a^	57 ± 11	54 ± 9	51 ± 10	61 ± 7 *	58 ± 9	54 ± 11	56 ± 10	56 ± 10
Height (cm) ^a^	174 ± 11	176 ± 8	176 ± 12	174 ± 8	173 ± 11	177 ± 9	175 ± 10	175 ± 10
Weight (kg) ^a^	90 ± 25	88 ± 22	96 ± 29	82 ± 14 *	85 ± 24	94 ± 23	86 ± 21	97 ± 29
NYHA stage (class II/III)	17/6	17/4	17/5	17/5	14/8	20/2	23/9	11/1
EF (%) ^b^	30 (25–40)	35 (28–38)	30 (25–41)	33 (30–35)	30 (25–39)	33 (29–40)	30 (25–35)	44 (40–45) *
**Baseline Cardiopulmonary Exercise Testing Indices**								
Peak VO_2_ (mL/kg/min) ^a^	15.1 ± 2.8	22.1 ± 2.3 *	16.2 ± 4.4	20.6 ± 3.2 *	17.3 ± 4.2	19.5 ± 4.4	18.6 ± 4.3	17.9 ± 4.6
Predicted peak VO_2_ (%) ^a^	55 ± 14	74 ± 11 *	52 ± 9	77 ± 8 *	63 ± 16	66 ± 15	65 ± 15	62 ± 18
VE/VCO_2_ slope ^a^	29 ± 6	29 ± 4	28 ± 5	30 ± 5	33 ± 4	25 ± 3 *	29 ± 5	28 ± 5
Peak WR (watts) ^a^	82 ± 33	122 ± 33 *	94 ± 41	108 ± 36	90 ± 38	112 ± 36	100 ± 39	104 ± 39

NYHA, New York Heart Association; EF, ejection fraction; VO_2_, oxygen uptake; VCO_2_, carbon dioxide output; WR, work rate. ^a^ Values are expressed as means ± SD. ^b^ Values are expressed as medians (25th–75th percentiles). * Difference between the 2 severity groups for variables regarding demographic characteristics or CPET parameters (*p* < 0.05).

**Table 2:** Differences in variables between patients with chronic heart failure of different severity, according to peak VO_2_, after a cardiac rehabilitation program.

	Peak VO_2_ ≤ 18.3 mL/kg/min 23 Patients		Peak VO_2_ > 18.3 mL/kg/min 21 Patients		*p*-Value between Groups
	Before CR	After CR	Before CR	After CR	
**Endothelial Cellular Populations ^b^**					
CD34^+^/CD45^−^/CD133^+^	54 (24–74)	98 (76–131) *	42 (20–71)	85 (50–112) **	0.213
CD34^+^/CD45^−^/CD133^+^/VEGFR_2_	2 (1–4)	7 (4–9) *	2 (1–3)	5 (3–7) **	0.055
CD34^+^/CD133^+^/VEGFR_2_	13 (9–16)	22 (17–36) **	10 (7–19)	23 (14–54) ***	0.125
CD34^+^/CD45^−^/CD133^−^	186 (131–287)	431 (301–618) **	234 (164–259)	520 (297–866) *	0.315
CD34^+^/CD45^−^/CD133^−^/VEGFR_2_	1 (1–3)	4 (3–8) **	1 (1–2)	5 (3–8) *	0.163
**Cardiopulmonary Exercise Testing Indices ^a^**					
Peak VO_2_ (mL/kg/min)	15.1 ± 2.8	18.4 ± 5.1 **	22.1 ± 2.3	23.3 ± 5.4	0.147
Predicted peak VO_2_ (%)	55 ± 14	67 ± 21 **	74 ± 11	79 ± 21	0.216
VE/VCO_2_ slope	29 ± 6	28 ± 6	29 ± 4	27 ± 5 **	0.566
Peak WR (watts)	82 ± 33	102 ± 38 *	122 ± 33	141 ± 43 *	0.731
**Blood Sample Indices ^b^**					
CRP (mg/dL)	0.2 (0.1–0.4)	0.1 (0.1–0.2) **	0.2 (0.1–0.6)	0.1 (0–0.4) **	0.798
IL-6 (pg/mL)	18.5 (15.9–23.5)	15.1 (13–22.2)	14.4 (12–18.6)	14.6 (11–18.3)	0.379
IL-10 (pg/mL)	24.5 (23.6–26.4)	24.7 (23.3–29.5)	24 (23.4–25.1)	25.9 (22.9–29.2)	0.642
VEGF (pg/mL)	14 (12–21)	20 (15–45) *	15 (13–19)	24 (20–35) *	0.235
EF (%) ^b^	30 (25–40)	35 (30–45) **	35 (28–38)	39 (30–43) **	0.802

CR, cardiac rehabilitation; VO_2_, oxygen uptake; VE, minute ventilation; VCO_2_, carbon dioxide output; WR, work rate; EF, ejection fraction; CRP, C-reactive protein; IL, interleukin; VEGF, vascular endothelial growth factor. ^a^ Values are expressed as means ± SD. ^b^ Values are expressed as medians (25th–75th percentiles). Differences within each severity group: * *p* < 0.001; ** *p* < 0.01; *** *p* < 0.05.

**Table 3:** Differences in variables between patients with chronic heart failure of different severity, according to predicted peak VO_2_, after a cardiac rehabilitation program.

	Predicted Peak VO_2_ ≤ 65.5% 22 Patients		Predicted Peak VO_2_ > 65.5% 22 Patients		*p*-Value between Groups
	Before CR	After CR	Before CR	After CR	
**Endothelial Cellular Populations ^b^**					
CD34^+^/CD45^−^/CD133^+^	50 (24–73)	97 (71–107) *	43 (22–85)	83 (48–120) *	0.624
CD34^+^/CD45^−^/CD133^+^/VEGFR_2_	2 (1–4)	6 (4–8) *	2 (1–3)	5 (3–8) *	0.368
CD34^+^/CD133^+^/VEGFR_2_	13 (8–16)	22 (15–41) ***	11 (7–18)	24 (14–38) **	0.120
CD34^+^/CD45^−^/CD133^−^	218 (128–259)	423 (297–575) **	201 (151–366)	542 (306–738) *	0.360
CD34^+^/CD45^−^/CD133^−^/VEGFR_2_	1 (1–3)	4 (3–9) **	1 (1–2)	5 (3–7) *	0.375
**Cardiopulmonary Exercise Testing Indices ^a^**					
Peak VO_2_ (mL/kg/min)	16.2 ± 4.4	19.2 ± 6.4 ***	20.6 ± 3.2	22.3 ± 4.6	0.368
Predicted peak VO_2_ (%)	52 ± 9	60 ± 16 **	77 ± 8	85 ± 20 ***	0.360
VE/VCO_2_ slope	28 ± 5	28 ± 6	30 ± 5	27 ± 5 **	0.977
Peak WR (watts)	94 ± 41	116 ± 49 *	108 ± 36	124 ± 41 *	0.087
**Blood Sample Indices ^b^**					
CRP (mg/dL)	0.4 (0.1–0.5)	0.1 (0.1–0.3) *	0.2 (0.1–0.4)	0 (0–0.2) **	0.678
IL-6 (pg/mL)	17.5 (13.7–23.5)	15.4 (12.9–21.4)	16.4 (12–21)	14.8 (11.8–18.3)	0.228
IL-10 (pg/mL)	24.3 (23.5–26.1)	24.4 (23.3–29.7)	24.2 (23.5–25.6)	26.8 (22.8–28.8) ***	0.757
VEGF (pg/mL)	13 (12–19)	20 (15–27) *	15 (13–20)	27 (20–63) *	0.116
EF (%) ^b^	30 (25–41)	35 (30–44) **	33 (30–35)	35 (30–45) **	0.717

CR, cardiac rehabilitation; VO_2_, oxygen uptake; VE, minute ventilation; VCO_2_, carbon dioxide output; WR, work rate; EF, ejection fraction; CRP, C-reactive protein; IL, interleukin; VEGF, vascular endothelial growth factor. ^a^ Values are expressed as means ± SD. ^b^ Values are expressed as medians (25th–75th percentiles). Differences within each severity group: * *p* < 0.001; ** *p* < 0.01; *** *p* < 0.05.

**Table 4:** Differences in variables between patients with chronic heart failure of different severity, according to VE/VCO_2_, after a cardiac rehabilitation program.

	VE/VCO_2_ > 28.1 22 Patients		VE/VCO_2_ ≤ 28.1 22 Patients		*p*-Value between Groups
	Before CR	After CR	Before CR	After CR	
**Endothelial Cellular Populations ^b^**					
CD34^+^/CD45^−^/CD133^+^	41 (20–66)	88 (53–98) *	51 (30–85)	104 (54–127) *	0.354
CD34^+^/CD45^−^/CD133^+^/VEGFR_2_	2 (1–4)	7 (4–9) *	2 (1–2)	5 (3–7) *	0.114
CD34^+^/CD133^+^/VEGFR_2_	12 (8–18)	23 (17–37) **	12 (7–17)	22 (14–45) **	0.760
CD34^+^/CD45^−^/CD133^−^	198 (144–380)	425 (284–768) **	218 (147–246)	452 (303–622) *	0.903
CD34^+^/CD45^−^/CD133^−^/VEGFR_2_	1 (1–2)	6 (4–9) **	1 (1–2)	4 (2–8) *	0.740
**Cardiopulmonary Exercise Testing Indices ^a^**					
Peak VO_2_ (mL/kg/min)	17.3 ± 4.2	19.5 ± 5.1	19.5 ± 4.4	22.0 ± 6.2 ***	0.880
Predicted peak VO_2_ (%)	63 ± 16	72 ± 20 ***	66 ± 15	74 ± 24 ***	0.853
VE/VCO_2_ slope	33 ± 4	31 ± 5	25 ± 3	24 ± 3	0.498
Peak WR (watts)	90 ± 38	108 ± 41 *	112 ± 36	133 ± 46 *	0.668
**Blood Sample Indices ^b^**					
CRP (mg/dL)	0.2 (0.1–0.4)	0.1 (0–0.2) **	0.2 (0.1–0.5)	0.1 (0–0.2) **	0.961
IL-6 (pg/mL)	17.4 (13.6–23.5)	15.7 (12.9–21.2)	16.4 (13.1–21.9)	14.8 (11.6–19)	0.253
IL-10 (pg/mL)	24.6 (23.5–26.1)	27.5 (23.4–30.4) ***	23.9 (23.5–25.1)	24.3 (22.9–27.6)	0.407
VEGF (pg/mL)	15 (13–21)	27 (18–70) *	14 (12–18)	21 (17–27) *	0.275
EF (%) ^b^	30 (25–40)	35 (29–44) **	35 (30–40)	40 (34–45) *	0.165

CR, cardiac rehabilitation; VO_2_, oxygen uptake; VE, minute ventilation; VCO_2_, carbon dioxide output; WR, work rate; EF, ejection fraction; CRP, C-reactive protein; IL, interleukin; VEGF, vascular endothelial growth factor. ^a^ Values are expressed as means ± SD. ^b^ Values are expressed as medians (25th–75th percentiles). Differences within each severity group: * *p* < 0.001; ** *p* < 0.01; *** *p* < 0.05.

**Table 5:** Differences in variables between patients with chronic heart failure of different severity, according to ejection fraction, after a cardiac rehabilitation program.

	EF < 40% 32 Patients		EF [40–49%] 12 Patients		*p*-Value between Groups
	Before CR	After CR	Before CR	After CR	
**Endothelial Cellular Populations ^b^**					
CD34^+^/CD45^−^/CD133^+^	45 (22–75)	88 (47–118) *	53 (38–71)	100 (79–118) **	0.618
CD34^+^/CD45^−^/CD133^+^/VEGFR_2_	2 (1–3)	5 (3–8) *	2 (1–4)	7 (4–8) **	0.743
CD34^+^/CD133^+^/VEGFR_2_	12 (7–18)	22 (14–37) **	12 (7–18)	31 (20–45) **	0.773
CD34^+^/CD45^−^/CD133^−^	201 (149–266)	437 (302–666) *	227 (135–334)	471 (253–772) **	0.858
CD34^+^/CD45^−^/CD133^−^/VEGFR_2_	1 (1–2)	5 (3–9) *	1 (1–2)	4 (4–8) **	0.596
**Cardiopulmonary Exercise Testing Indices ^a^**					
Peak VO_2_ (mL/kg/min)	18.6 ± 4.3	20.6 ± 5.8 ***	17.9 ± 4.6	21.1 ± 5.9 ***	0.459
Predicted peak VO_2_ (%)	65 ± 15	72 ± 21 ***	62 ± 18	74 ± 23 **	0.422
VE/VCO_2_ slope	29 ± 5	28 ± 6	28 ± 5	26 ± 5	0.906
Peak WR (watts)	100 ± 39	120 ± 44 *	104 ± 39	121 ± 47 ***	0.647
**Blood Sample Indices ^b^**					
CRP (mg/dL)	0.2 (0.1–0.5)	0.1 (0–0.2) *	0.1 (0.1–0.4)	0.1 (0–0.3) **	0.706
IL-6 (pg/mL)	16.8 (13.4–20.4)	14.8 (12.2–18.1)	16.6 (12.5–23.5)	20.6 (12.8–22.8)	0.067
IL-10 (pg/mL)	24.4 (23.6–26.3)	25 (23.1–29.9)	23.7 (23.5–24.7)	24.8 (23.1–28.2)	0.150
VEGF (pg/mL)	14 (13–20)	22 (17–37) *	15 (12–19)	24 (16–65) **	0.886
EF (%) ^b^	30 (25–35)	35 (30–35) *	44 (40–45)	45 (44–50) ***	0.726

CR, cardiac rehabilitation; VO_2_, oxygen uptake; VE, minute ventilation; VCO_2_, carbon dioxide output; WR, work rate; EF, ejection fraction; CRP, C-reactive protein; IL, interleukin; VEGF, vascular endothelial growth factor. ^a^ Values are expressed as means ± SD. ^b^ Values are expressed as medians (25th–75th percentiles). Differences within each severity group: * *p* < 0.001; ** *p* < 0.01; *** *p* < 0.05.

**Table 6:** Linear regression analysis between the absolute values and the percentages Δθ of each EPC and CEC subgroup after the cardiac rehabilitation program and the baseline values of peak VO_2_, predicted peak VO_2_, VE/VCO_2_ slope, EF, and age.

	Peak VO_2_	Predicted Peak VO_2_	VE/VCO_2_ Slope	Ejection Fraction	Age
**Absolute Δθ**					
CD34^+^/CD45^−^/CD133^+^	−1.39 (−15.95, 13.15)	−0.20 (−4.53, 4.13)	1.33 (−2.39, 5.07)	0.54 (−1.55, 2.64)	−0.24 (−5.09, 4.59)
CD34^+^/CD45^−^/CD133^+^/VEGFR_2_	−0.55 (−2.72, 1.62)	0.13 (−0.51, 0.77)	0.28 (−0.27, 0.84)	0.10 (−0.21, 0.41)	−0.18 (−0.90, 0.53)
CD34^+^/CD133^+^/VEGFR_2_	−0.97 (−8.98, 7.02)	0.39 (−1.98, 2.78)	0.73 (−1.31, 2.78)	−0.03 (−1.19, 1.11)	−0.42 (−3.08, 2.23)
CD34^+^/CD45^−^/CD133^−^	−60.91 (−224.99, 103.15)	9.48 (−39.42, 58.39)	18.40 (−23.67, 60.48)	−12.17 (−35.78, 11.44)	−23.83 (−78.43, 30.76)
CD34^+^/CD45^−^/CD133^−^/VEGFR_2_	−0.09 (−2.06, 1.87)	0.04 (−0.54, 0.63)	−0.02 (−0.52, 0.48)	−0.27 (−0.56, 0.01)	0.09 (−0.55, 0.75)
**Percentage Δθ**					
CD34^+^/CD45^−^/CD133^+^	−36.60 (−171.75, 98.53)	5.45 (−34.83, 45.73)	19.77 (−14.88, 54.43)	3.81 (−15.64, 23.26)	−17.56 (−62.53, 27.40)
CD34^+^/CD45^−^/CD133^+^/VEGFR_2_	−40.37 (−147.25, 66.50)	7.83 (−24.02, 39.69)	21.13 (−6.27, 48.54)	2.84 (−12.54, 18.22)	−6.64 (−42.20, 28.92)
CD34^+^/CD133^+^/VEGFR_2_	33.03 (−206.57, 272.64)	−7.04 (−78.46, 64.37)	26.14 (−35.30, 87.59)	−8.77 (−43.25, 25.71)	9.60 (−70.12, 89.33)
CD34^+^/CD45^−^/CD133^−^	18.27 (−386.84, 423.40)	−14.17 (−134.93, 106.58)	62.95 (−40.95, 166.85)	7.86 (−50.45, 66.17)	−29.44 (−164.25, 105.35)
CD34^+^/CD45^−^/CD133^−^/VEGFR_2_	12.24 (−79.94, 104.43)	−7.57 (−35.05, 19.90)	−13.93 (−37.57, 9.71)	−12.70 (−25.97, 0.56)	14.78 (−15.89, 45.45)

Note: *p* > 0.05 for all variables.

## Data Availability

Data will be made available upon request.

## Supplementary Materials

The following supporting information can be downloaded at: https://www.mdpi.com/article/10.3390/jcm12206592/s1, Table S1: Correlations between the numeric differences in the mobilization of endothelial cellular populations and the numeric differences in other cardiopulmonary exercise testing or blood sample variables after the cardiac rehabilitation program; Table S2: Correlations between the percentage differences in the mobilization of endothelial cellular populations and the percentage differences in other cardiopulmonary exercise testing or blood sample variables after the cardiac rehabilitation program.
