# Supplementary material for: The Effects of a Cardiac Rehabilitation Program on Endothelial Progenitor Cells and Inflammatory Profile in Patients with Chronic Heart Failure of Different Severity
Source: J Clin Med. 2023 Oct 18;12(20):6592. doi: 10.3390/jcm12206592 (PMC10607596; doi:10.3390/jcm12206592)
Supplement: Supplementary file 1 [file jcm-12-06592-s001.zip › jcm-2641483-supplementary.pdf]

Supplementary Table S1. Correlations between the numeric difference of the mobilization of endothelial cellular populations and the numeric difference of other cardiopulmonary exercise testing or blood sample variables after the cardiac rehabilitation program

	**Peak VO_2**	**Predicted peak VO_2**	**VE/VCO2 slope**	**Peak WR**	**CRP**	**IL-6**	**IL-10**	**VEGF**
**$CD34^+/CD45^-/CD133^+$**	0.063 (CC)<br>p = 0.686	0.042 (CC)<br>p = 0.786	0.038 (CC)<br>p = 0.807	-0.008 (CC)<br>p = 0.957	0.002 (CC)<br>p = 0.991	-0.034 (CC)<br>p = 0.827	-0.149 (CC)<br>p = 0.336	0.100 (CC)<br>p = 0.518
**$CD34^+/CD45^-/CD133^+/VEGFR_2$**	-0.273 (CC)<br>p = 0.073	-0.237 (CC)<br>p = 0.121	-0.007 (CC)<br>p = 0.967	-0.165 (CC)<br>p = 0.284	-0.090 (CC)<br>p = 0.561	-0.168 (CC)<br>p = 0.276	-0.067 (CC)<br>p = 0.667	0.029 (CC)<br>p = 0.851
**$CD34^+/CD133^+/VEGFR_2$**	-0.167 (CC)<br>p = 0.280	-0.196 (CC)<br>p = 0.203	0.007 (CC)<br>p = 0.962	-0.059 (CC)<br>p = 0.702	0.284 (CC)<br>p = 0.061	-0.264 (CC)<br>p = 0.083	-0.154 (CC)<br>p = 0.317	-0.115 (CC)<br>p = 0.455
**$CD34^+/CD45^-/CD133^-$**	-0.047 (CC)<br>p = 0.761	-0.012 (CC)<br>p = 0.938	0.170 (CC)<br>p = 0.270	0.085 (CC)<br>p = 0.584	-0.059 (CC)<br>p = 0.705	-0.080 (CC)<br>p = 0.607	-0.192 (CC)<br>p = 0.212	**-0.309 (CC)**<br>**p = 0.041**
**$CD34^+/CD45^-/CD133^-/VEGFR_2$**	0.244 (CC)<br>p = 0.111	0.271 (CC)<br>p = 0.075	-0.018 (CC)<br>p = 0.906	0.251 (CC)<br>p = 0.101	0.288 (CC)<br>p = 0.058	0.039 (CC)<br>p = 0.801	0.028 (CC)<br>p = 0.856	0.120 (CC)<br>p = 0.436

CR, cardiac rehabilitation; **VO_2**, oxygen uptake; **VE**, minute ventilation; **VCO_2**, carbon dioxide output; **WR**, work rate; **CRP**, C-reactive protein; **IL**, interleukin; **VEGF**, vascular endothelial growth factor.

Supplementary Table S2. Correlations between the percentage difference of the mobilization of endothelial cellular populations and the percentage difference of other cardiopulmonary exercise testing or blood sample variables after the cardiac rehabilitation program

	**Peak VO_2**	**Predicted peak VO_2**	**VE/VCO2 slope**	**Peak WR**	**CRP**	**IL-6**	**IL-10**	**VEGF**
**$CD34^+/CD45^-/CD133^+$**	-0.018 (CC)<br>p = 0.906	-0.057 (CC)<br>p = 0.716	0.263 (CC)<br>p = 0.084	-0.020 (CC)<br>p = 0.898	-0.030 (CC)<br>p = 0.849	0.014 (CC)<br>p = 0.926	-0.006 (CC)<br>p = 0.968	0.273 (CC)<br>p = 0.073
**$CD34^+/CD45^-/CD133^+/VEGFR_2$**	-0.177 (CC)<br>p = 0.250	-0.148 (CC)<br>p = 0.338	0.105 (CC)<br>p = 0.496	0.041 (CC)<br>p = 0.791	-0.085 (CC)<br>p = 0.583	-0.084 (CC)<br>p = 0.588	-0.085 (CC)<br>p = 0.582	0.046 (CC)<br>p = 0.766
**$CD34^+/CD133^+/VEGFR_2$**	0.255 (CC)<br>p = 0.095	0.242 (CC)<br>p = 0.113	-0.042 (CC)<br>p = 0.784	0.168 (CC)<br>p = 0.276	0.161 (CC)<br>p = 0.296	-0.142 (CC)<br>p = 0.359	-0.164 (CC)<br>p = 0.288	-0.162 (CC)<br>p = 0.293
**$CD34^+/CD45^-/CD133^-$**	-0.136 (CC)<br>p = 0.378	-0.103 (CC)<br>p = 0.506	0.197 (CC)<br>p = 0.200	0.010 (CC)<br>p = 0.951	0.044 (CC)<br>p = 0.774	0.007 (CC)<br>p = 0.963	-0.192 (CC)<br>p = 0.212	-0.261 (CC)<br>p = 0.088
**$CD34^+/CD45^-/CD133^-/VEGFR_2$**	**0.402 (CC)**<br>**p = 0.007**	**0.379 (CC)**<br>**p = 0.011**	-0.017 (CC)<br>p = 0.914	**0.333 (CC)**<br>**p = 0.027**	0.073 (CC)<br>p = 0.638	0.190 (CC)<br>p = 0.218	0.206 (CC)<br>p = 0.179	0.139 (CC)<br>p = 0.369

CR, cardiac rehabilitation; **VO_2**, oxygen uptake; **VE**, minute ventilation; **VCO_2**, carbon dioxide output; **WR**, work rate; **CRP**, C-reactive protein; **IL**, interleukin; **VEGF**, vascular endothelial growth factor.
